# Construction of a SNP-Based High-Density Genetic Map Using Genotyping by Sequencing (GBS) and QTL Analysis of Nut Traits in Chinese Chestnut (*Castanea mollissima* Blume)

**DOI:** 10.3389/fpls.2018.00816

**Published:** 2018-06-14

**Authors:** Feiyang Ji, Wei Wei, Yang Liu, Guangpeng Wang, Qing Zhang, Yu Xing, Shuhang Zhang, Zhihao Liu, Qingqin Cao, Ling Qin

**Affiliations:** ^1^Department of Plant Science and Technology, Beijing Key Laboratory of Agricultural Application and New Technique, Beijing University of Agriculture, Beijing, China; ^2^Changli Institute of Pomology, Hebei Academy of Agriculture and Forestry Sciences, Changli, China; ^3^Beijing Collaborative Innovation Center for Eco-Environmental Improvement with Forestry and Fruit Trees, Beijing, China; ^4^Novogene Bioinformatics Technology Co., Ltd., Tianjin, China; ^5^Department of Biological Science and Engineering, Key Laboratory of Urban Agriculture (North China), Ministry of Agriculture, Beijing University of Agriculture, Beijing, China

**Keywords:** *Castanea mollissima*, genetic map, genotyping by sequencing, single nucleotide polymorphism, QTL, nut traits

## Abstract

Chinese chestnut is a wildly distributed nut species with importantly economic value. The nut size and ripening period are mainly desired breeding objectives in Chinese chestnut. However, high-density linkage maps and quantitative trait loci (QTL) analyses related to nut traits are less than satisfactory, which hinders progress in the breeding of Chinese chestnut. Here, a single nucleotide polymorphism (SNP)-based high-density linkage map was constructed through genotyping-by-sequencing (GBS) of an F_1_ cross between the two widely grown Chinese chestnut cultivars ‘Yanshanzaofeng’ and ‘Guanting No. 10’. The genetic linkage map consists of 2,620 SNP markers with a total length of 1078.06 cM in 12 linkage groups (LGs) and an average marker distance of 0.41 cM. 17 QTLs were identified for five nut traits, specifically single-nut weight (SNW), nut width (NW), nut thickness (NT), nut height (NH), and ripening period (RP), based on phenotypic data from two successive years. Of the 17 QTLs, two major QTLs, i.e., *qNT-I-1* and *qRP-B-1* related to the NT and RP traits, respectively, were exploited. Moreover, the data revealed one pleiotropic QTL at 23.97 cM on LG I, which might simultaneously control SNW, NT, and NW. This study provides useful benchmark information concerning high-density genetic mapping and QTLs identification related to nut size and ripening period, and will accelerate genetic improvements for nuts in the marker-assisted selection (MAS) breeding of Chinese chestnut.

## Introduction

*Castanea* is one of the most economically and ecologically important genera in the Fagaceae family. Three *Castanea* species, i.e., the Chinese chestnut (*C*. *mollissima*), Japanese chestnut (*C. crenata*) and European chestnut (*C. sativa*), are widely cultivated for commercial nut production (Huang, 1998). Due to the high nutritional value of edible nuts, the chestnut, which is generally considered a woody grain, plays a significant role in human famine history. The chestnut also plays an important ecological role in afforestation and ecosystem services (Martin et al., 2012; Zou et al., 2014). There are diverse germplasm resources for Chinese chestnut, and this plant has a long history of cultivation. The nut yield of Chinese chestnut ranks first worldwide, with an annual production of 1,650,000 tons^1^, accounting for 82% of the total chestnut production (Barreira et al., 2008; Liang et al., 2009; Zhang et al., 2014).

Due to its excellent nut quality, good adaptability to adverse environments, high resistance to main diseases and easily peeled pellicle (Bounous and Marinoni, 2005; Nishio et al., 2013), Chinese chestnut has been broadly used as a breeding parent to improve nut quality and disease resistance (Kubisiak et al., 1997, 2013; Sisco et al., 2005). However, conventional breeding and the juvenile period (3–5 years) impede progress in chestnut breeding compared with annual plants. Marker-assisted selection (MAS) approaches can efficiently solve these problems. Genetic maps and quantitative trait loci (QTLs) have been developed as essential tools to assist MAS programs in many plant species (Wheeler and Sederoff, 2009; Wu J. et al., 2014; Chen et al., 2015; Ren et al., 2016; Jiang et al., 2017; Zhao et al., 2017).

Several different types of molecular markers have been used to construct genetic maps, such as isozymes, restriction fragment length polymorphisms (RFLPs), randomly amplified polymorphic DNA (RAPD), amplified fragment length polymorphisms (AFLPs), simple sequence repeats (SSRs) and single nucleotide polymorphisms (SNPs) (Kubisiak et al., 1997, 2013; Casasoli et al., 2001; Sisco et al., 2005). Among them, SNPs have been increasingly used for the construction of high-density genetic maps for many crops (Chagné et al., 2007; Barba et al., 2014; Talukder et al., 2014; Wu G.A. et al., 2014; Bielenberg et al., 2015; Ipek et al., 2016; Su et al., 2017) due to their high abundance and relatively even distribution across a genome. Moreover, genotyping-by-sequencing (GBS) approaches, which utilize NGS technologies, can generate a high number of SNP markers, and the use of these SNP markers could allow the construction of high-density genetic maps (Ward et al., 2013; Chen et al., 2014; Gardner et al., 2014; Guajardo et al., 2015; Soto et al., 2015).

Over the last 20 years, several genetic maps of the *Castanea* genus had been constructed, and several QTLs were identified using these genetic maps. The first *Castanea* genetic map was constructed using an F_2_ population of the interspecific cross between the American and Chinese chestnut species and consisted of 196 RAPD and RFLP markers covering 530.10 cM (Kubisiak et al., 1997). Using this map, Kubisiak et al. (1997) proposed a three-QTL model that explained approximately 70% of the phenotypic variance in resistance against blight. Casasoli et al. (2004) constructed a genetic map containing 217 markers (142 RAPDs, three isozymes, 30 ISSRs, and 42 SSRs) with an average distance of 8.7 cM using an F_1_ population of 152 individuals and identified QTLs related to bud flush, growth, and carbon isotope discrimination. Several genetic maps were subsequently developed, but these maps did not further extend the densities (Casasoli et al., 2001; Sisco et al., 2005; Guo et al., 2008; Hu, 2008). The most recently constructed genetic map for the *Castanea* genus contains 329 SSR and 1,064 SNP markers derived from expressed sequence tags (ESTs) integrated with a physical map (Fang et al., 2013; Kubisiak et al., 2013) and allowed identification of three QTL regions involved in the resistance to chestnut blight. The density of this genetic map was increased, but the number of markers was still lower than that of other crops (Li et al., 2014; Zhao et al., 2014; Ipek et al., 2016; Zhang et al., 2016). Because the fruit/nut has important economic value and the fruit/nut quality is considered the primary selection criteria by breeders of fruit crops, several QTLs correlated with important fruit quality traits, such as the fruit shape, sugar content, acid content, maturity, and fruit skin composition, have been studied for various fruit crops, including peach (Martínez-García et al., 2013), apple (Kenis et al., 2008; Dunemann et al., 2009; Chagne et al., 2012), strawberry (Zorrilla-Fontanesi et al., 2011), sweet cherry (Zhang et al., 2010), apricot (Campoy et al., 2011; Socquet-Juglard et al., 2013), and papaya (Blas et al., 2012), hawthorn (Zhao et al., 2017). However, the mapping of QTLs related chestnut nut traits has not been reported. High-density genetic maps and QTLs identification are urgently required to achieve excellent nut traits of chestnut through MAS breeding.

At present, one of the major problems in chestnut production in China is the high proportion of late-ripening cultivars, which has resulted in a concentrated market supply of fresh nuts and a decline in prices. Early ripening cultivars not only satisfy consumer demands but also show a great price superiority; as a result, these cultivars meet the requirements of chestnut growers and compensate for the insufficiency of the early chestnut supply. One of the main purpose of the breeding of Chinese chestnut is to supply the early chestnut market with cultivars that mature early and present good nut quality. Therefore, in this study, we selected a well-established F_1_ population derived from two widely grown Chinese chestnut cultivars, i.e., ‘Yanshanzaofeng’ (ripens early at the beginning of September) and ‘Guanting No. 10’ (ripens late at the end of September), to construct a high-density genetic linkage map based on SNP markers using GBS. Moreover, by combining this high-density genetic map with phenotypic data of the cross parents and F_1_ progenies collected over two successive years, the QTLs for nut traits, including the single-nut weight (SNW), nut width (NW), nut thickness (NT), nut height (NH) and ripening period (RP), were characterized. The high-density genetic map and identified QTLs add invaluable knowledge for genetic and MAS research, which would facilitate the breeding of new varieties and germplasms with good agronomic nut traits.

## Materials and Methods

### Mapping Population and DNA Extraction

The F_1_ mapping population consisted of 259 progenies generated by crossing ‘Yanshanzaofeng’ and ‘Guanting No. 10’. The F_1_ progeny seeds were germinated and grown in the spring of 2012, and the seedlings were then transplanted to the Chestnut Germplasm Resources Station at the Changli Institute of Pomology, Hebei Academy of Agriculture and Forestry Sciences.

Young leaves of the 259 F_1_ individuals and the parents were harvested, immediately frozen in liquid nitrogen and maintained at -80°C. The genomic DNA was extracted using a modified cetyltrimethylammonium bromide (CTAB) method (Cheng et al., 2005).

### Library Preparation and Illumina Sequencing

First, we performed a GBS pre-design experiment and evaluated the enzymes and sizes of the restriction fragments using the following three criteria: (i) the number of tags must be suitable for the specific needs of the research project; (ii) the enzymatic tags must be evenly distributed throughout the genome; and (iii) repeated tags should be avoided. These considerations improved the efficiency of the GBS. To maintain uniformity of the sequence depth in the different fragments, a strict length range was selected (∼50 bp).

Second, a GBS library was constructed using a pre-designed scheme. For the F_1_ population, the genomic DNA was incubated at 37°C with *Mse*I (New England Biolabs, NEB, United States), T4 DNA ligase (NEB, United States), ATP (NEB, United States), and an *Mse*I Y-adapter N-containing barcode. The restriction-ligation reactions were heat-inactivated at 65°C and then digested with the *Hae*III (GGCC) restriction enzyme at 37°C. The restriction-ligation samples were purified using Agencourt AMPure XP (Beckman, United States). Polymerase chain reaction (PCR) was conducted using the purified samples, the Phusion Master Mix (NEB, United States) universal and index primers, and i5 and i7 sequences. The PCR products were purified using Agencourt AMPure XP, pooled, and electrophoresed on a 2% agarose gel. A Gel Extraction Kit (Qiagen, Germany) was used to isolate 375-to-400-bp fragments (with indexes and adaptors). These fragments were purified using Agencourt AMPure XP, and the resulting products were diluted for sequencing.

Then, pair-end sequencing of the selected tags was performed using an Illumina high-throughput sequencing platform at the Novogene Bioinformatics Technology Company, China, and SNP genotyping and evaluation were then performed.

### Quality Assessment

The sequences of each sample were sorted according to their barcodes. To ensure that the reads used in the subsequent analyses were reliable without an artificial bias (low-quality paired reads, which mainly resulted from base-calling duplicates and adapter contamination), the raw data (raw reads in fastq format) were processed using a series of in-house C scripts for quality control (QC). During the QC procedures, the following reads were removed: (i) reads with ≥10% unidentified nucleotides (N); (ii) reads with >50% bases with a phred quality < 5; (iii) reads with >10-nt alignment to the adapter, allowing ≤10% mismatches; and (iv) reads that contain the *Hae*III enzyme sequence.

### SNP Discovery and Genotyping

Burrows-Wheeler Aligner (BWA) (Li and Durbin, 2009) software was used to align the clean reads of each sample against the Chinese chestnut genome^2^. The alignment files were converted to bam files using SAMtools software (Li et al., 2009). If multiple read pairs had identical external coordinates, only the pair with the highest mapping quality was retained. Variant calling was performed for all samples using GATK software (McKenna et al., 2010). The SNPs were then filtered using a Perl script, and ANNOVAR (Wang et al., 2010) was used to annotate the SNPs based on the GFF3 files of the reference genome.

All SNP markers between the parents were classified into eight segregation patterns (ab × cd, ef × eg, hk × hk, lm × ll, nn × np, aa × bb, ab × cc, and cc × ab). For the F_1_ population, the segregation patterns <hk × hk>, <lm × ll> and <nn × np> were selected for construction of the genetic map. The polymorphic heterozygous SNP markers in only one of the parents were scored as <lm × ll> or <nn × np>, and the heterozygous markers in both parents were scored as <hk × hk>. The ratios of the marker segregation were calculated using a chi-square test, and only the markers that satisfied the expected Mendelian segregation ratio (*p* > 0.001) were included in the mapping.

### Genetic Map Construction

Markers containing segregation distortions (*p* < 0.001), missing more than 15% of the data or containing abnormal bases were excluded from the map construction. The ‘two-way pseudo-test cross’ approach (Grattapaglia and Sederoff, 1994) was used to construct the genetic linkage map. For the linkage analysis, JoinMap^®^ 4.0 software was used to sort the markers in each linkage group (LG) (van Ooijen, 2006). A logarithm of odds (LOD) threshold of 2.0–25 was used to determine the LGs. Maternal, paternal and integrated maps were constructed using regression-based parameters and the Kosambi mapping function was used to calculate the genetic distance between the markers (Kosambi, 1944).

### Phenotypic Data Collection

Nuts from the F_1_ population and the two parents were collected at maturity, and five phenotypic traits, namely, the RP, NW, NT, NH, and SNW, were measured (**Supplementary Figure S1**). The RP was calculated from the time at which catkin became fully open to the 30% bur cracking per tree. The width, thickness and height of the nuts were measured using a digital caliper, and the SNW was recorded in grams. In total, 30 nuts collected from each tree in 2015 and 2016 were used for the determination of phenotypic data. The average values of each trait per individual were used in the QTL analysis.

### Statistical Analysis

The phenotypic data were analyzed using SPSS 22.0 software (SPSS, United States) to generate descriptive statistics, including the mean, minimum, maximum, standard deviation (SD), coefficient of variation (CV), skewness and kurtosis. The frequency distribution of phenotypic data were checked using SPSS 22.0 software as well. The kurtosis and skewness were used to estimate the frequency distribution normality (Tisne et al., 2008), and the correlations among the SNW, NW, NT, NH, and RP traits were analyzed using two-tailed bivariate correlation tests.

### Analysis of QTLs

Quantitative trait loci analysis of the five nut traits for the Chinese chestnut was performed using the interval mapping (IM) model in MapQTL 6.0 (Van Ooijen, 2009). The threshold LOD at a significance level of *P* = 0.05 for each trait was calculated based on 1,000 permutations. The QTLs with an LOD above the threshold were deemed significant. The phenotypic variance explained (PVE) of a single QTL was estimated based on a maximum likelihood estimation, and those with a PVE ≥ 15% were considered major QTLs. All of the identified QTLs were drawn on LGs using a Perl SVG module.

## Results

### Genotyping by Sequencing

We obtained ∼111.10 G of high-quality raw sequencing data. After data trimming and filtering, ∼1.66 G of high-quality data were generated for the parents, with an average of ∼830.39 M, and ∼109.37 G of high-quality data were generated for the progenies, with an average of ∼422.27 M. The Q30 ratio was 91.76%, and the guanine-cytosine (GC) content was 36.11%. In total, 379,747,764 clean reads were obtained from 259 individuals, 2,799,642 clean reads were obtained from the maternal parent, ‘Yanshanzaofeng,’ and 2,966,960 clean reads were obtained from the paternal parent, ‘Guanting No. 10’. The number of clean reads obtained from the 261 samples ranged from 1.30 to 5.90 M, with an average of 2.95 M reads (**Supplementary Figure S2**), and 95.71% of the clean reads were matched with the Chinese chestnut genome. As shown in **Supplementary Figure S3**, the 1× coverage in the F_1_ individuals and the parents ranged from 4.74 to 11.6%, with an average of 7.62%, and the 4× coverage ranged from 1.56 to 5.37 %, with an average of 3.38%.

### SNP Marker Detection and Genotyping

After data filtering, 62,348 SNP loci were retained between the parents, and of these, 49,049 were transition-type and transversion-type SNPs. The transition-type SNPs accounted for 35.37% (17, 351) and 35.86% (17, 591) of the C/T and A/G types, respectively, and the other four SNP types were transversions and included A/C, G/T, A/T and C/G, ranging from 4.94% (2, 421) to 8.88% (4, 356) as shown in **Table 1**. Because the genetic background of the two parents was heterozygous, three marker segregation type codes, i.e., <lm × ll>, <nn × np> (providing 1:1 segregation ratios) and <hk × hk> (providing a segregation ratio of 1:2:1), were used to score the heterozygous loci in the female and male parents and both parents, respectively. After integrity filtering and a chi-square test, 3,719 SNP markers were retained for further analyses. Of these, 1,407 heterozygous SNPs were found in ‘Yanshanzaofeng’ (lm × ll), 1,503 heterozygous SNPs were found in ‘Guanting No. 10’ (nn × np), and the remaining 809 heterozygous SNPs were found in both parents (hk × hk). After the filtration of completeness degree and partial separation, 3719 markers were obtained for linkage grouping. However, in the process of linkage grouping using JoinMap^®^ 4.0 software, some of the markers that are not linked can not enter into the last 12 LG, therefore, 2620 SNP markers were retained for the construction of the genetic map after linkage determination.

**Table 1:** Statistics of the identified SNP marker types.

Type of variation	Number	Proportion of type %
A/G	17,591	35.86
C/T	17,351	35.37
A/T	4,356	8.88
C/G	2,421	4.94
A/C	3,630	7.40
G/T	3,700	7.54
Total	49,049	100.00

### Linkage Map Construction

**FIGURE 1:**
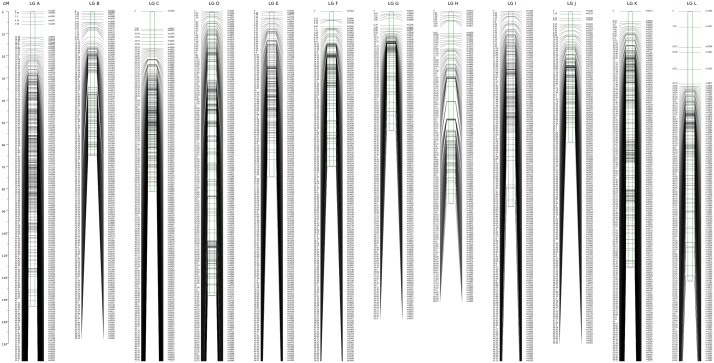
Integrated LGs in the Chinese chestnut using the ‘Yanshanzaofeng’ × ‘Guanting No. 10’ cross.

The 2,620 SNP markers were assigned to 12 LGs (LG A–LG L) spanning a length of 1078.06 cM, with an average distance of 0.41 cM between adjacent markers in the integrated map (**Figure 1** and **Table 2**). The average distance between adjacent markers i L) (**Table 3**). We constructed two high-density genetic maps from the cross of ‘Yanshanzaofeng’ and ‘Guanting No. 10’ using JoinMap^®^ 4.0. The male parent ‘Guanting No. 10’ consisted of 1,318 SNP markers in 12 LGs, and the length of the ‘Guanting No. 10’ map was 768.86 cM, with an average distance of 0.58 cM between adjacent markers. However, the average distance between adjacent markers ranged from 0.31 cM (LG C) to 1.36 cM (LG H). The female parent, ‘Yanshanzaofeng,’ consisted of 1,769 markers in 12 LGs with a map length of 750.52 cM, and the average interval between adjacent mapped markers of 0.42 cM ranged from 0.32 cM (LG C) to 2.46 cM (LG L). In all three maps, the longest LG was LG A (133.17 cM), and the shortest was LG F (33.22 cM).

**Table 2:** Summary of the mapping results in the Chinese chestnut genetic maps.

	Number of markers				Genetic size (cM)		
	Female	Male	Integrated map		Female	Male	Integrated map
LG A	223	234	434		80.36	112.14	133.17
LG B	124	53	149		47.99	37.14	65.13
LG C	229	142	290		73.98	44.06	81.22
LG D	144	141	282		52.40	63.01	128.46
LG E	166	89	199		75.31	40.63	74.75
LG F	159	73	167		69.14	33.22	69.94
LG G	127	61	139		44.72	52.83	53.90
LG H	122	46	131		55.63	62.53	86.72
LG I	123	120	199		48.53	79.56	88.16
LG J	128	74	149		44.80	41.81	59.04
LG K	190	137	305		73.88	111.08	115.78
LG L	34	148	176		83.78	90.85	121.79
Total	1769	1318	2620		750.52	768.86	1078.06

**Table 3:** Distribution of the SNP markers in the integrated genetic linkage groups.

Linkage Group	Average distance between two makers (cM)	Number of intervals—D^a^ (cM)				
		D < 1	1<5	D<5	5<D<10	D>10
LG A	0.31	407	24	431	2	0
LG B	0.44	138	9	147	1	0
LG C	0.28	276	12	288	1	0
LG D	0.46	254	27	281	0	0
LG E	0.38	186	11	197	1	0
LG F	0.42	149	17	166	0	0
LG G	0.39	121	17	138	0	0
LG H	0.66	109	18	127	3	0
LG I	0.44	182	14	196	1	1
LG J	0.40	133	14	147	1	0
LG K	0.38	275	29	304	0	0
LG L	0.69	147	23	170	5	0
Total	0.41	2377	215	2592	15	1

^a^*D, distance between adjacent markers*.

**FIGURE 2:**
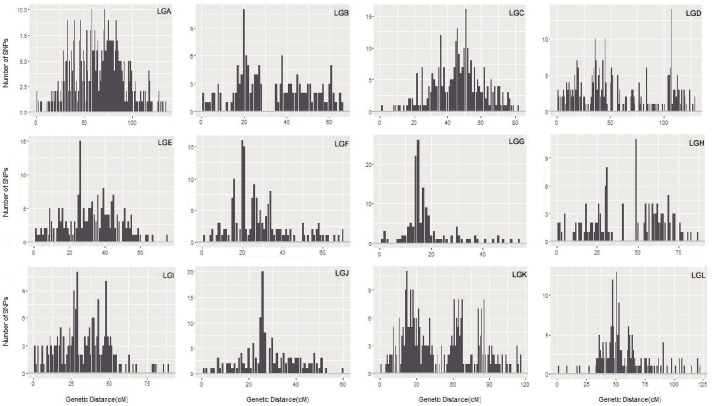
The marker interval positioning in each LG. The *x*-axis indicates the position in each LG in 1 cM intervals, and the *y*-axis indicates the number of markers within 1 cM.

The maximum number of markers in the ‘Yanshanzaofeng’ map was 229 in LG C, the maximum number of markers in the ‘Guanting No. 10’ map was 234 in LG A, and of the maximum number of markers in the integrated map was 305 in LG K. The minimum number of markers found in the ‘Yanshanzaofeng’ map was 34 in LG L, the minimum number of markers in the ‘Guanting No. 10’ map was 46 in LG H, and the minimum number of markers in the integrated map was 131 in LG H. Most of the SNP markers were evenly distributed in the 12 LGs (**Table 3**). Of the 2,608 intervals between adjacent markers in the 12 different LGs, 2,377 intervals were less than 1 cM, and 215 intervals were within 1 cM < D < 5 cM. Fifteen intervals with a distance of 5 cM < D < 10 cM between adjacent markers were observed in eight LGs, including LG A (2), LG B (1), LG C (1), LG E (1), LG H (3), LG I (1), LG J (1), and LG L (5) (**Figure 2** and **Table 3**).

### Phenotypic Analysis

The nut traits of F_1_ population exhibited wide segregation or variation (**Supplementary Figure S4**). Values for the mean, minimum, maximum, SD, CV, skewness and kurtosis were calculated for the five nut phenotypic traits (**Supplementary Table S1**), and all of the traits showed a normal distribution in both years studied. Moreover, the kurtosis and skewness of the phenotypic data for all five traits were less than 2. These results indicated that all traits underwent quantitative inheritance and were controlled by multiple genes (Zhang et al., 2013). The SNW, NT, NW, and NH showed highly significant correlations with each other (**Table 4**). The SNW showed a positive significant correlation with the NH, NT and NW, with correlation coefficients of 0.801^∗∗^, 0.825^∗∗^, and 0.659^∗∗^, respectively. The RP was not correlated with the SNW, NT or NW, but was significantly correlated (*R* = 0.136^∗^) with the NH (**Table 4**).

**Table 4:** Pearson correlation coefficients for different nut traits in the ‘Yanshanzaofeng’ × ‘Guanting No. 10’ F_1_ population.

Trait	Single-nut weight (g)	Nut thickness (mm)	Nut width (mm)	Nut height (mm)	Ripening period (days)
Single-nut weight	–	0.801^∗∗^	0.825^∗∗^	0.659^∗∗^	0.018
Nut thickness	–	–	0.637^∗∗^	0.454^∗∗^	-0.069
Nut width	–	–	–	0.609^∗∗^	0.023
Nut height	–	–	–	–	0.136^∗^
Ripening period	–	–	–	–	–

^∗^*P < 0.05, ^∗∗^*P* < 0.01*.

### QTLs for Nut Traits

**FIGURE 3:**
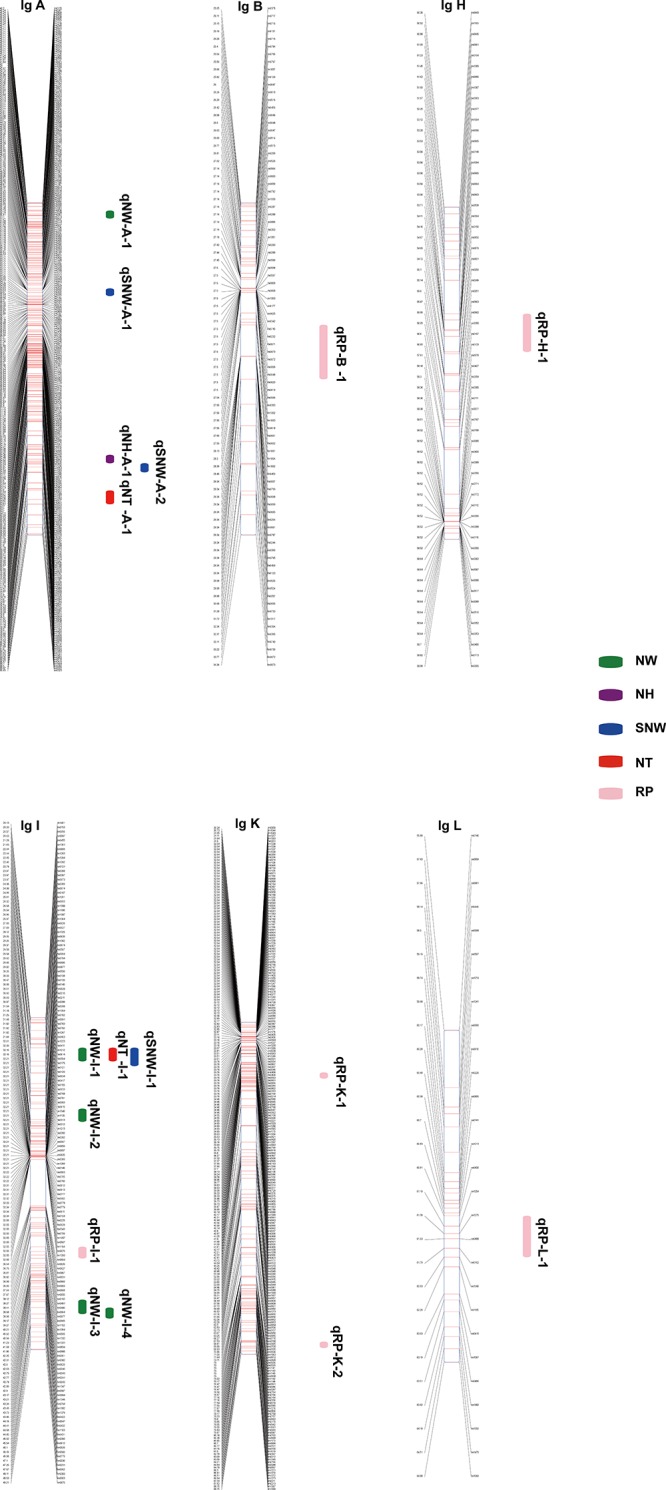
Quantitative trait loci position on the LGs. SNW QTLs are shown in blue, NW QTLs are shown in green, NT QTLs are shown in red, NH QTLs are shown in purple, and RP QTLs are shown in pink.

In total, 17 QTLs for SNW, NT, NW, NH and RP were identified based on the integrated genetic map of Chinese chestnut and were located on LG A, LG B, LG H, LG I, LG K, and LG L (**Figure 3** and **Table 5**). The phenotypic variance ranged from 9.1 to 16%, and the LOD values ranged from 3.65 to 4.93%. Moreover, two QTLs, i.e., *qNT-I-1* and *qRP-B-1* (PVE ≥ 15%), were considered major QTLs. In addition, we found two clusters of QTLs located on LG A and LG I.

**Table 5:** Quantitative trait loci analysis of Chinese chestnut nut traits in the F_1_ population.

Trait	Year	QTL	LG	Marker interval	Position (cM)	LOD	PVE (%)
SNW	2015	*qSNW-A-2*	A	nn0606-nn0098	79.12	4.57	11.1
		*qSNW-I-1*	I	hk0398-hk0073	23.97	4.07	9.9
	2016	*qSNW-A-1*	A	lm1207-nn0199	53.17	3.97	13.7
		*qSNW-I-1*	I	hk0397-hk0399	23.97	3.65	12.7
NT	2015	*qNT-A-1*	A	nn1451-nn0016	84.35	4.06	10
		*qNT-I-1*	I	hk0398-hk0073	23.97	4.93	12
	2016	*qNT-I-1*	I	hk0397-hk0399	23.97	4.71	16
NW	2015	*qNW-I-1*	I	hk0398-hk0073	23.97	4.45	11
		*qNW-I-4*	I	lm0912-nn0582	46.1	5	12.3
	2016	*qNW-A-1*	A	lm0137-nn1065	41.84	3.92	13.7
		*qNW-I-2*	I	lm1326-lm1382	29.05	3.77	13.2
		*qNW-I-3*	I	lm0431-lm0912	45.02	4.01	13.9
NH	2015	*qNH-A-1*	A	lm1153-nn0099	78.96	4.68	11.4
RP	2015	*qRP-I-1*	I	lm0505-nn1331	40.94	3.66	9.1
		*qRP-L-1*	L	nn1275-nn0162	61.53	4.73	11.6
	2016	*qRP-H-1*	H	lm0096-hk0149	53.53	4.19	14.1
		*qRP-K-1*	K	hk0376-lm0319	39.33	3.67	12.5
		*qRP-K-2*	K	lm1372-lm0211	86.64	3.67	12.5
		*qRP-B-1*	B	hk0699-lm0254	29.34	4.47	15

*LG, linkage group; Position, position on LG; LOD, logarithm of odds; PVE, phenotypic variance explained by QTL*.

Three QTLs were identified for SNW, and of these, *qSNW-I-1* appeared to be located on LG I in a region centered at 23.97 cM in the two successive years studied, with a phenotypic variance of 9.9% in 2015 and 12.7% in 2016. The other two QTLs, i.e., *qSNW-A-1* and *qSNW-A-2*, were located on LG A, were centered at 53.17 and 79.12 cM, and explained 13.7% (2016) and 11.1% (2015) of the phenotypic variance, respectively. We detected two QTLs for NT on LG A and LG I. *qNT-A-1*, which was centered at 84.35 cM, was only identified in 2015 and accounted for 10% of the variation, and *qNT-I-1*, which was centered at 23.97 cM, accounted for 12 and 16% of the variation in 2015 and 2016, respectively.

Five QTLs associated with the NW were identified. Of these QTLs, *qNW-I-1* and *qNW-I-4* were found on LG I and accounted for 11 and 12.3%, respectively, of the phenotypic variance in 2015. *qNW-A-1*, *qNW-I-2* and *qNW-I-3* were located on LG A and LG I in the integrated map and accounted for 13.7, 13.2, and 13.9% of the variance, respectively, in 2016. The QTL *qNH-A-1* for the NH was detected on LG A and accounted for 11.4% of the observed phenotypic variance in 2015. No QTLs were identified for 2016.

Six QTLs related to the RP were identified for the 2 years and were localized in different LGs. For 2015, *qRP-I-1* and *qRP-L-1* were identified on LG I and LG L at 40.94 and 61.53 cM, respectively, and explained 9.1 and 11.6% of the phenotypic variance, respectively. However, for 2016, *qRP-H-1*, *qRP-K-1*, *qRP-K-2*, and *qRP-B-1* were found on LG H, LG K and LG B, centered at 53.53, 39.33, 86.84, and 29.34 cM and accounted for 14.1, 12.5, 12.5 and 15% of the phenotypic variance, respectively.

In addition, a single QTL associated with each of the traits of SNW, NT and NW was identified in the same position, specifically at 23.97 cM in LG I, which might be related to a single locus with pleiotropic effects.

## Discussion

### Construction of High-Density Genetic Map

Eight genetic linkage maps have been developed to date for *Castanea* (**Table 6**). However, these genetic maps were mainly constructed using RAPD, RFLP and inter-SSR (ISSR) markers and did not achieve a significant improvement in map density, with the exception of the genetic map described by Kubisiak et al. (2013). Kubisiak et al. (2013) first constructed a high-density map of Chinese chestnut using 1,064 SNP markers and 329 SSR markers derived from a database of ESTs in Fagaceae. This map was the first genetic map for *Castanea* with more than 1000 markers and a mean interval between adjacent markers of <1.0 cM (0.70 cM). In the present study, mapping using markedly larger numbers of markers (2,620 SNP markers mapped) was achieved with GBS sequencing. Furthermore, the genetic linkage map constructed in this study has the shortest average genetic interval (0.41 cM/marker) and thus constitutes the highest-density genetic map for *Castanea* plants constructed to date. In our study the length of integrated genetic map, which spans 1,078.06 cM, was longer than that obtained in previous studies. The number of genetic markers (2620 markers) used in this study was far more than that used in the construction of previous maps. The primary reasons for the larger number of genetic markers in this study might be the highly heterozygous genetic background of the hybrid population and the larger population size (261), which resulted in an increased allele complexity (Fang et al., 2013). So far, there are several genetic maps used LG 1–12 or A-L LG identifiers. It is necessary to make consistent each other. 129 common SNPs were found in both genetic LGs between this study and Kubisiak et al. (2013), and each LG in both genetic LGs has common SNP markers to consist with Kubisiak et al. (2013) (**Supplementary Table S2**). Chinese chestnut was used as the mapping parents for six of the nine *Castanea* genetic maps (**Table 6**), which is consistent with its favorable traits, including disease resistance, good nut quality and respectable adaptability.

**Table 6:** Comparison of the linkage maps in *Castanea* genus.

No.	Species	Name of parent	Number of progenies	Number of markers	Total of markers	Map length (cM)	Average distance (cM)	LG	References
1	*C. mollissima* ×*C. dentata*	R4T52 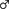 × R4T31 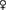	F_2_(102)	RAPDs(170), isozymes(2), RFLPs(12)	184	530.10	2.80	12	Kubisiak et al., 1997
2	*C. sativa*	Bursa 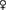 × Hopa 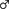	F_1_(96)	RAPDs(311), ISSRs(65), isozymes(5)	187 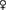	720	9.00	12	Casasoli et al., 2001
					148 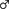	721	8.70		
3	*C. sativa*	Bursa 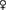 × Hop 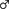 a	F_1_(152)	RAPDs(142), isozymes(3), ISSRs(30), SSRs(42)	109 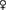	848.60	8.70	12	Casasoli et al., 2004
					108 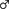	832.90	8.70		
4	*C. mollissima* ×*C. dentata*	R4T52 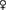 × R4T31 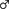	F_2_(193)	isozymes(1), AFLPs(275), SSRs(24)	300	551.10	–	12	Sisco et al., 2005
5	*C. mollissim*a	‘Baopi’ 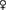 × ‘Chuizhi’ 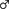	F_1_(60)	RAPD	88 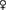	823.10	9.40	10	Guo et al., 2008
					55 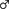	720.80	13.10	7	
6	*C. mollissima*		F_1_	RAPDs	105	841.10	8.01	12	Hu, 2008
7	*C. mollissima*	‘Mahogany’ 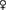 × ‘Nanking’ 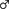	F_1_(179)	SSRs(329), SNPs(1064)	1393	742.40	0.70	12	Kubisiak et al., 2013
		‘Vanuxem’ 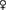 × ‘Nanking’ 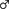	F_1_(158)						
8	C. *crenata*	‘Tanzawa 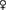 × Porotan 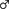 ’	F_1_(47)	SSRs(11)	11	–	–	–	Nishio et al., 2013
		‘550-40 (P/p) 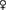 × Tanzawa 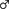 ’	F_1_(69)						
9	*C. mollissima*	‘Yanshanzaofeng’ 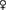 × ‘Guanting No. 10’ 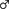	F_1_(259)	SNPs(2620)	2620	1078.06	0.41	12	Present study

### Analysis of QTLs for Fruit/Nut Traits

Quantitative trait loci for fruit traits play an important role in breeding programs and have been reported for many crops, such as plum (Salazar et al., 2017), pea (Ma et al., 2017), and coffee (Moncada et al., 2016). However, a limited number of QTLs have been identified for *Castanea* plants. Kubisiak et al. (2013) identified three QTLs for resistance against blight, and Casasoli et al. (2004) detected QTLs for agronomic traits of the *Castanea* genus. In our study, 17 QTLs were identified for five nut traits based on 2-year phenotypic data. Of these, five QTLs aggregated on LG A, and seven QTLs clustered on LG I. The clustering of genes with similar functions was universal (Sun et al., 2009), as was observed for peach (Eduardo et al., 2011) and papaya (Blas et al., 2012). Additionally, previous studies noted that QTLs for highly correlated traits mapped to the same or adjacent LG regions (Zhang et al., 2013; Chang et al., 2014). In this study, the SNW was highly correlated with the NT and NW, and *qSNW-I-1* for SNW also shared genetic loci at 23.97 cM with *qNT-I-1* for NT and *qNW-I-1* for NW. We speculated that the traits related to *qSNW-I-1*, *qNT-I-1* and *qNW-I-1* might be controlled by one QTL. This phenomenon can be explained by the existence of a single locus with pleiotropic effects (Eduardo et al., 2011).

Stable QTLs for different years are very valuable and practical for MAS breeding programs and have been identified in many species (Chang et al., 2014; Wu et al., 2015). Two stable QTLs for fruit quality traits were identified in apple (Potts et al., 2014). Zhang et al. (2013) identified a stable QTL for timing of fruit maturity in pear based on a 2-year study. These stable QTLs could be used in early selection for molecular breeding. In our study, several QTLs for SNW, NT and NW were found to be stable in two successive years, and of these QTLs, *qNT-I-1* was considered a major QTL. These stable and major QTLs could be useful in MAS approaches to facilitate the genetic improvement of Chinese chestnut. However, the authenticity of a QTL must be tested in different environments and different mapping populations (Paterson et al., 1991). For the RP trait, no stable QTLs were detected in our 2-year study. Moreover, QTLs for NH were detected in 2015 but not in 2016, primarily due to the influence of the environment on the NH and RP traits.

## Conclusion

A high-density linkage map of Chinese chestnut was constructed using SNP markers obtained through GBS sequencing. The genetic linkage map comprised 2620 SNP markers covering 1078.06 cM, and the average distance between adjacent markers was 0.41 cM. Based on this genetic map, 17 QTLs were identified for five nut traits, including three QTLs for the SNW, two QTLs for the NT, five QTLs for the NW, one QTL for the NH and six QTLs for the RP. Moreover, two major QTLs related to the NT and RP were identified in successive 2 years.

## Data Statement

The raw sequencing data for individual samples has been deposited in NCBI-SRA and is accessible through the BioProject number PRJNA349111. The SNP data has uploaded on NCBI dbSNP (ss numbers: 2137339710 and 2629806925–2632384009).

## Author Contributions

LQ, QC, and GW: Conceived and designed the experiments. FJ, WW, YL, and SZ: Performed the experiments. FJ, WW, and ZL: Analyzed the data. FJ, WW, and QC: Wrote the manuscript. QC, LQ, YX, and QZ: Read and approved the final manuscript.

## Conflict of Interest Statement

The authors declare that the research was conducted in the absence of any commercial or financial relationships that could be construed as a potential conflict of interest.

## Footnotes

1 http://faostat3.fao.org

2 http://www.hardwoodgenomics.org/chinese-chestnut-genome

## Supplementary Material

FIGURE S1 Nut parameters including width, thickness, and height.

FIGURE S2 Distribution of read numbers in F_1_ individuals. The *x*-axis indicates the interval of the read number, and the *y*-axis indicates the number of individuals.

FIGURE S3 **(A,B)** Coverage of each plant in the F_1_ population and their parents. The *x*-axis shown in **(A,B)** indicates the plant accession, including the two parents and their average, the *y*-axis indicates the 1× coverage of the reads in **(A)** and 4× coverage of the reads in **(B)**.

FIGURE S4 Nut phenotype of parents (‘Yanshanzaofeng’ and ‘Guanting No. 10’) and six individuals of F_1_ population. Female parent, ‘Yanshanzaofeng’; male parent, ‘Guanting No. 10’. Six individuals are YG12-82, YG9-12, YG13-24, YG12-160, YG9-1, and YG10-80, respectively.

TABLE S1 Data on nut traits collected from the F_1_ population of ‘Yanshanzaofeng’ × ‘Guanting No. 10’ in two successive years (2015 and 2016).

TABLE S2 Related linkage groups of ‘Yanshanzaofeng’ × ‘GuantingNo. 10’, and ‘Vanuxem’ × ‘Nanking’.
