# Supplementary figures and images for: Construction of a SNP-Based High-Density Genetic Map Using Genotyping by Sequencing (GBS) and QTL Analysis of Nut Traits in Chinese Chestnut (Castanea mollissima Blume)
Source: Front Plant Sci. 2018 Jun 14;9:816. doi: 10.3389/fpls.2018.00816 (PMC6011034; doi:10.3389/fpls.2018.00816)

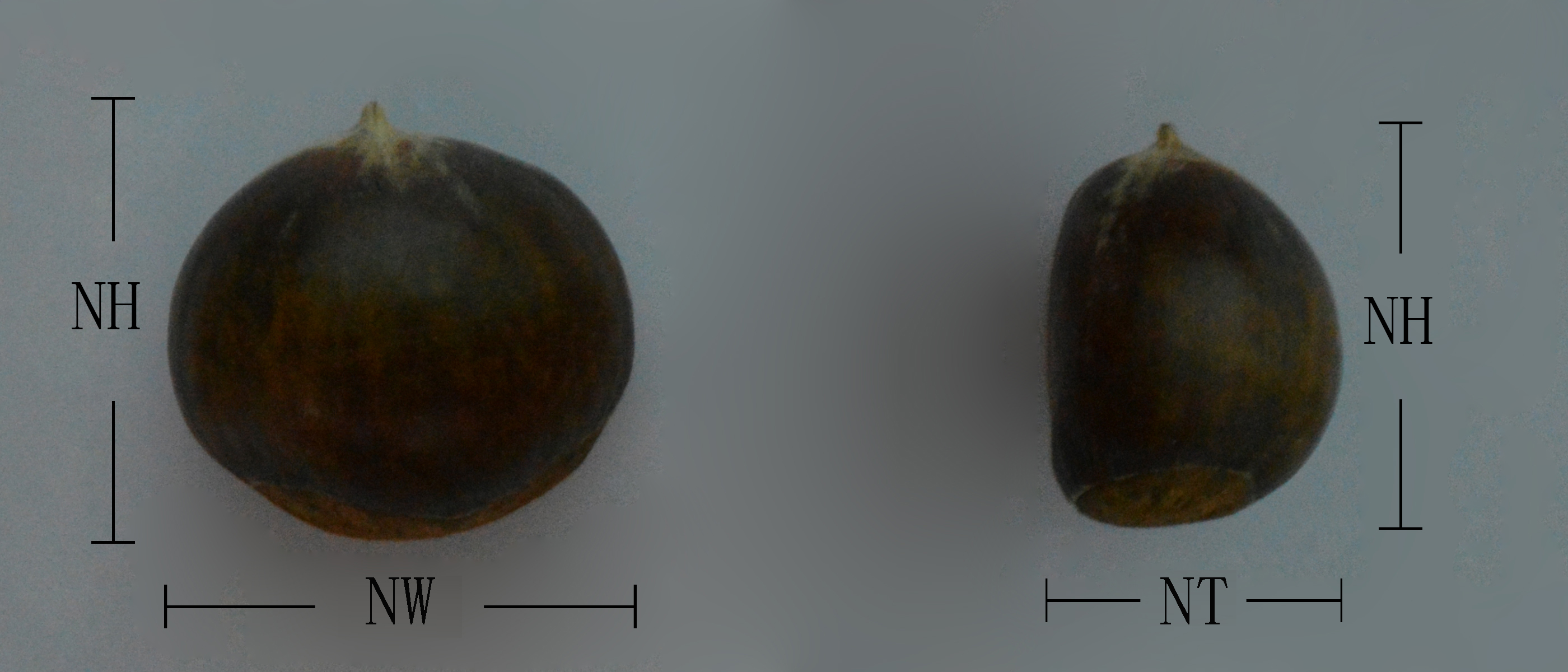

Supplement: FIGURE S1 — Nut parameters including width, thickness, and height. [file Image_1.JPEG]

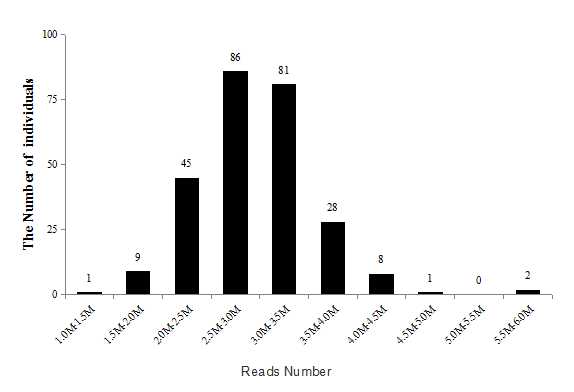

Supplement: FIGURE S2 — Distribution of read numbers in F1 individuals. The x-axis indicates the interval of the read number, and the y-axis indicates the number of individuals. [file Image_2.JPEG]

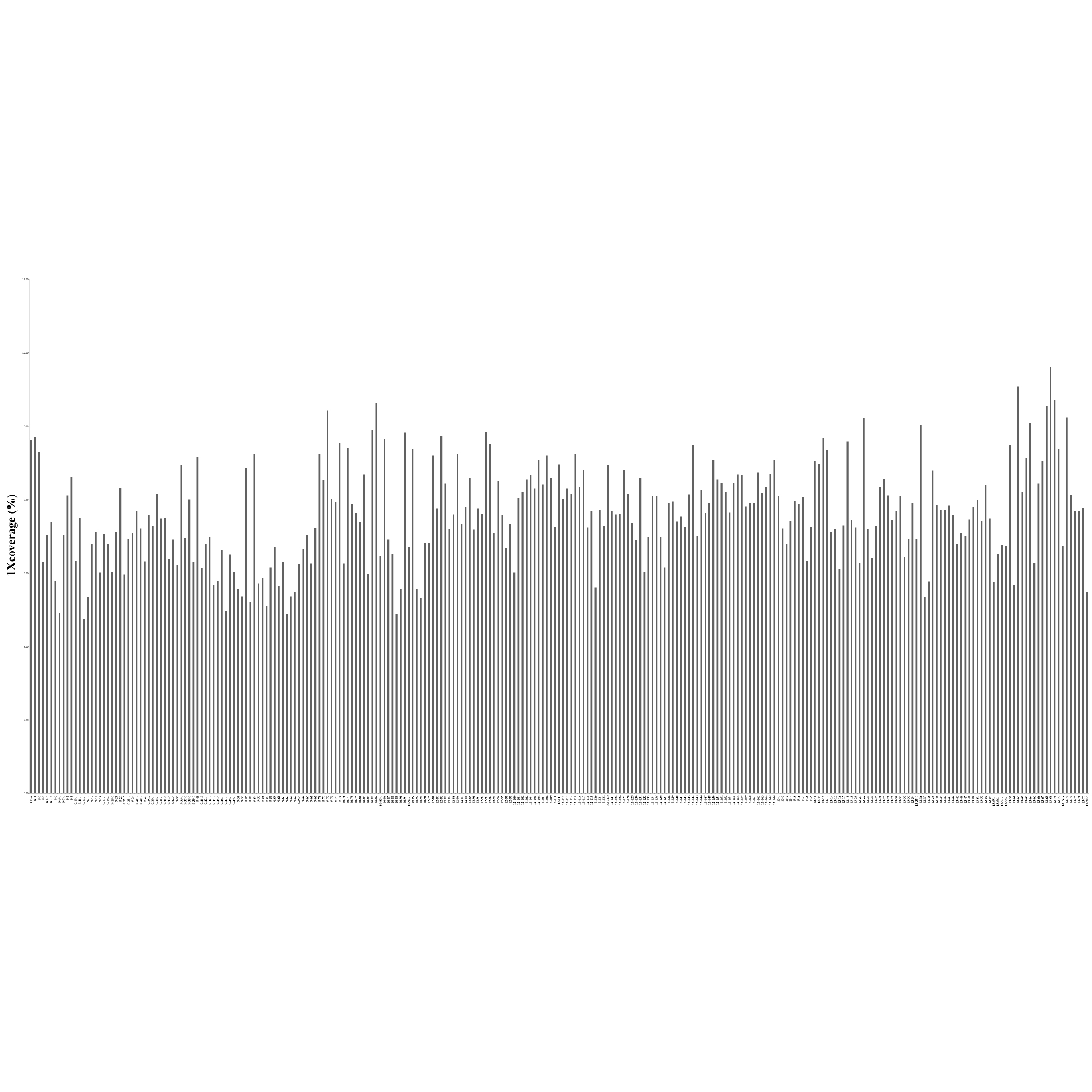

Supplement: FIGURE S3 — (A,B) Coverage of each plant in the F1 population and their parents. The x-axis shown in (A,B) indicates the plant accession, including the two parents and their average, the y-axis indicates the 1× coverage of the reads in (A) and 4× coverage of the reads in (B). [file Image_3.JPEG]

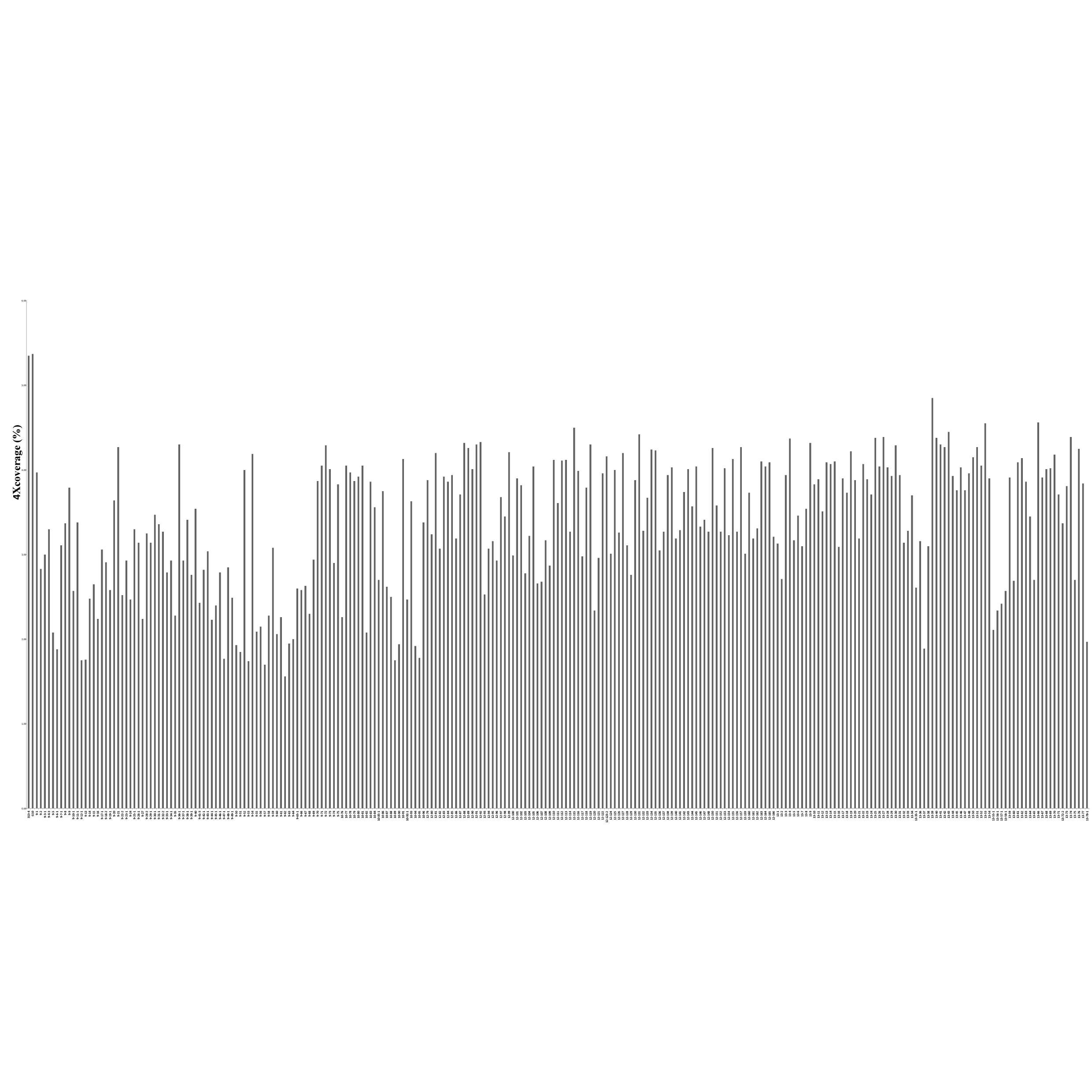

Supplement: FIGURE S4 — Nut phenotype of parents (‘Yanshanzaofeng’ and ‘Guanting No. 10’) and six individuals of F1 population. Female parent, ‘Yanshanzaofeng’; male parent, ‘Guanting No. 10’. Six individuals are YG12-82, YG9-12, YG13-24, YG12-160, YG9-1, and YG10-80, respectively. [file Image_4.JPEG]

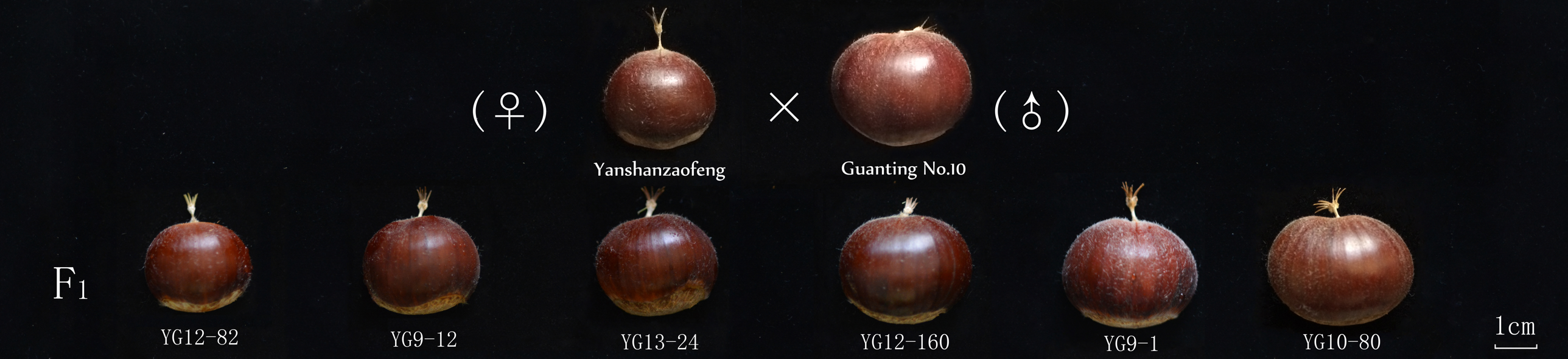

Supplement: Supplementary file 5 [file Image_5.JPEG]
