# Supplementary material for: Construction of a SNP-Based High-Density Genetic Map Using Genotyping by Sequencing (GBS) and QTL Analysis of Nut Traits in Chinese Chestnut (Castanea mollissima Blume)
Source: Front Plant Sci. 2018 Jun 14;9:816. doi: 10.3389/fpls.2018.00816 (PMC6011034; doi:10.3389/fpls.2018.00816)
Supplement: TABLE S1 — Data on nut traits collected from the F1 population of ‘Yanshanzaofeng’ × ‘Guanting No. 10’ in two successive years (2015 and 2016). [file Table_1.docx]

Construction of a SNP-based High-density Genetic Map Using Genotyping by Sequencing (GBS) and QTL Analysis of Nut Traits in Chinese Chestnut (*Castanea mollissima* Blume)

Ji Feiyang^1^, Wei Wei^1^, Liu Yang^1^, Wang Guangpeng^2^, Zhang Qing^1^, Xing Yu^1,3^, Zhang Shuhang^2^, Liu Zhihao^4^, Cao Qingqin^3,5*^, Qin Ling^1,3*^

*** Correspondence:**

Cao Qingqin

[caoqingqin@sina.com](mailto:caoqingqin@sina.com)

Qin Ling

[qinlingbac@126.com](mailto:qinlingbac@126.com)

Ji Feiyang and Wei Wei contributed equally to this work.

**Supplemental Table 1.** Data on nut traits collected from an F_1_ population of ‘Yanshanzaofeng’ × ‘Guanting No.10’ in two successive years (2015 and 2016).

|  | Single-nut weight (g) | Nut thickness  (mm) | Nut width  (mm) | Nut height  (mm) | ripening period  (days) |
| --- | --- | --- | --- | --- | --- |
| Mean | 8.24/9.21^1^ | 18.10/19.37 | 28.25/29.11 | 24.15/24.84 | 96.70/96.51 |
| Minimum | 4.80/4.70 | 14.05/14.40 | 18.55/22.98 | 18.53/20.43 | 87.00/85.00 |
| Maximum | 12.70/12.36 | 21.79/22.94 | 34.15/34.60 | 27.80/31.60 | 115.00/112.00 |
| S.D. | 1.27/1.31 | 1.30/1.43 | 2.01/1.83 | 1.28/1.45 | 5.33/6.03 |
| C.V. | 0.15/0.14 | 0.07/0.07 | 0.07/0.06 | 0.05/0.06 | 0.06/0.06 |
| Skewness | 0.31/0.02 | 0.23/-0.26 | -0.75/-0.09 | -0.54/0.97 | 0.71/0.45 |
| Kurtosis | 0.60/0.55 | 0.29/0.65 | 1.2/0.88 | 1.4/1.3 | 0.25/-0.22 |

^1^2015 data / 2016 data
