# Supplementary material for: Construction of a SNP-Based High-Density Genetic Map Using Genotyping by Sequencing (GBS) and QTL Analysis of Nut Traits in Chinese Chestnut (Castanea mollissima Blume)
Source: Front Plant Sci. 2018 Jun 14;9:816. doi: 10.3389/fpls.2018.00816 (PMC6011034; doi:10.3389/fpls.2018.00816)
Supplement: TABLE S2 — Related linkage groups of ‘Yanshanzaofeng’ × ‘GuantingNo. 10’, and ‘Vanuxem’ × ‘Nanking’. [file Table_2.DOCX]

Construction of a SNP-based High-density Genetic Map Using Genotyping by Sequencing (GBS) and QTL Analysis of Nut Traits in Chinese Chestnut (*Castanea mollissima* Blume)

Ji Feiyang^1^, Wei Wei^1^, Liu Yang^1^, Wang Guangpeng^2^, Zhang Qing^1^, Xing Yu^1,3^, Zhang Shuhang^2^, Liu Zhihao^4^, Cao Qingqin^3,5*^, Qin Ling^1,3*^

*** Correspondence:**

Cao Qingqin

[caoqingqin@sina.com](mailto:caoqingqin@sina.com)

Qin Ling

[qinlingbac@126.com](mailto:qinlingbac@126.com)

Ji Feiyang and Wei Wei contributed equally to this work.

**Supplemental Table 2.** Related linkage groups of ‘Yanshanzaofeng’×‘GuantingNo.10’, and ‘Vanuxem’×‘Nanking’

| ‘Yanshanzaofeng’♀×‘GuantingNo.10’♂Linkage Groups | ‘Vanuxem’♀×‘Nanking’♂Linkage Groups | markers-in-common |
| --- | --- | --- |
| LG A | A | hk0003/CmSNP00002^1^, hk0549/CmSNP00044, nn1117/CmSNP00059, nn0988/P00095, lm0369/CmSNP00116, lm0649/CmSNP00191, lm0260/CmSP00281, lm0837/CmSP00613, nn1405/CmSP00652, lm1326/CmSP00655, nn1148/CmSP00739, hk0056/CmSP00759, lm0005/CmSP00831, hk0291/CmSP00939, nn0871/CmSP01112, nn1019/CmSP01161, lm0601/CmSP01209, lm0147/CmSP01255, nn0802/CmSP01400 |
| LG B | B | hk0700/CmSNP00046, lm0730/CmSNP00458, hk0007/CmSNP00527, lm0516/CmSNP00660 |
| LG C | C | lm0630/CmSNP00063, lm1084/CmSNP00180, nn0886/CmSNP00194, nn0260/CmSNP00223, lm0367/CmSNP00279, lm0478/CmSNP00307, lm0124/CmSNP00365, hk0055/CmSNP00596, hk0439/CmSNP00659, nn0138/CmSNP00891, lm0972/CmSNP01053, lm1000/CmSNP01121, nn0867/CmSNP01289, hk0471/CmSNP01524 |
| LG D | D | nn0053/CmSNP00070, nn0011/CmSNP00218, nn0028/CmSNP00478, nn0880/CmSNP01365, nn1003/CmSNP01387, nn1121/CmSNP01395, nn0437/CmSNP01457, nn0900/CmSNP01463 |
| LG E | E | lm1374/CmSNP00021, hk0182/CmSNP00140, hk0401/CmSNP00671, hk0084/CmSNP00955, lm0497/CmSNP00984, lm1355/CmSNP01106, lm0479/CmSNP01444 |
| LG F | F | nn0602/CmSNP00084, hk0681/CmSNP00161, nn0649/CmSNP00294, lm0285/CmSNP00427, lm0862/CmSNP00504, lm0178/CmSNP00688, hk0557/CmSNP01220, hk0532/CmSNP01368, lm1297/CmSNP01485 |
| LG G | G | lm0889/CmSNP00415, nn0346/CmSNP00689, hk0671/CmSNP00700, nn0450/CmSNP00884, hk0763/CmSNP00910, lm0889/P00415, nn0346/P00689, hk0671/P00700, nn0450/P00884, hk0763/P00910 |
| LG H | H | lm0983/CmSNP00091, lm1067/CmSNP00839, lm0650/CmSNP00870, lm0983/P00091, lm1067/P00839, lm0650/P00870 |
| LG I | I | lm0876/CmSNP00078, hk0398/CmSNP00170, lm0967/CmSNP00247, hk0507/CmSNP00299, lm1235/CmSNP00605, lm0876/P00078, hk0398/P00170, lm0967/P00247, hk0507/P00299, lm1235/P00605, hk0417/P00706, lm0357/P01070, nn0070/P01111, hk0594/P01482 |
| LG J | J | lm0350/CmSNP00033, lm0039/CmSNP00037, lm1375/CmSNP00232, lm0543/CmSNP00241, lm0822/CmSNP00255, lm0350/P00033, lm0039/P00037, lm1375/P00232, lm0543/P00241, lm0822/P00255, lm0280/P00474, nn0922/P01215, nn1173/P01291 |
| LG K | K | lm0728/CmSNP00211, lm1199/CmSNP00408, hk0618/CmSNP01356, lm1316/CmSNP01409, lm0728/P00211, lm1199/P00408, hk0618/P01356, lm1316/P01409 |
| LG L | L | nn0018/CmSNP00130, nn0140/CmSNP00215, hk0014/CmSNP00284, nn0816/CmSNP00377, nn1364/CmSNP00376, nn0018/P00130, nn0140/P00215, hk0014/P00284, nn1364/P00376, nn0816/P00377, lm0420/P00803, nn0746/P00829, nn1151/P00869, lm0759/P00935, P01243/P01243, nn0036/P01405, nn1274/P01506 |

**^1^** ‘Yanshanzaofeng’×‘GuantingNo.10’ / ‘Vanuxem’×‘Nanking’
